# Robust Assessment of EEG Connectivity Patterns in Mild Cognitive Impairment and Alzheimer's Disease

**DOI:** 10.3389/fnimg.2022.924811

**Published:** 2022-07-11

**Authors:** Ruaridh A. Clark, Keith Smith, Javier Escudero, Agustín Ibáñez, Mario A. Parra

**Affiliations:** ^1^Centre for Signal and Image Processing, University of Strathclyde, Glasgow, United Kingdom; ^2^Department of Physics and Mathematics, Nottingham Trent University, Nottingham, United Kingdom; ^3^School of Engineering, Institute for Digital Communications, University of Edinburgh, Edinburgh, United Kingdom; ^4^Latin American Brain Health Institute, Universidad Adolfo Ibáñez, Santiago, Chile; ^5^Cognitive Neuroscience Center, Universidad de San Andrés & Consejo Nacional de Investigaciones Científicas y Técnicas (CONICET), Buenos Aires, Argentina; ^6^Global Brain Health Institute (GBHI), University of California, San Francisco, CA, United States; ^7^Global Brain Health Institute (GBHI), Trinity College, Dublin, Ireland; ^8^Trinity College Institute of Neuroscience, Trinity College Dublin, Dublin, Ireland; ^9^School of Psychological Sciences and Health, University of Strathclyde, Glasgow, United Kingdom

**Keywords:** EEG, coherency, connectivity, eigenvector, network topological analysis

## Abstract

The prevalence of dementia, including Alzheimer's disease (AD), is on the rise globally with screening and intervention of particular importance and benefit to those with limited access to healthcare. Electroencephalogram (EEG) is an inexpensive, scalable, and portable brain imaging technology that could deliver AD screening to those without local tertiary healthcare infrastructure. We study EEG recordings of subjects with sporadic mild cognitive impairment (MCI) and prodromal familial, early-onset, AD for the same working memory tasks using high- and low-density EEG, respectively. A challenge in detecting electrophysiological changes from EEG recordings is that noise and volume conduction effects are common and disruptive. It is known that the imaginary part of coherency (iCOH) can generate functional connectivity networks that mitigate against volume conduction, while also erasing true instantaneous activity (zero or π-phase). We aim to expose topological differences in these iCOH connectivity networks using a global network measure, eigenvector alignment (EA), shown to be robust to network alterations that emulate the erasure of connectivities by iCOH. Alignments assessed by EA capture the relationship between a pair of EEG channels from the similarity of their connectivity patterns. Significant alignments—from comparison with random null models—are seen to be consistent across frequency ranges (delta, theta, alpha, and beta) for the working memory tasks, where consistency of iCOH connectivities is also noted. For high-density EEG recordings, stark differences in the control and sporadic MCI results are observed with the control group demonstrating far more consistent alignments. Differences between the control and pre-dementia groupings are detected for significant correlation and iCOH connectivities, but only EA suggests a notable difference in network topology when comparing between subjects with sporadic MCI and prodromal familial AD. The consistency of alignments, across frequency ranges, provides a measure of confidence in EA's detection of topological structure, an important aspect that marks this approach as a promising direction for developing a reliable test for early onset AD.

## 1. Introduction

AD is considered to be a brain disconnection syndrome (Delbeuck et al., 2003), where earlier studies confirmed that brain connectivity, as assessed via cerebral coherence, significantly correlates with the clinical progression of the disease (Calderon et al., 1997). More recent studies have gone on to confirm that such a disconnection can be detected in the very early pre-dementia stages (Parra et al., 2017; Spyrou et al., 2018; Josefsson et al., 2019). Historically, quantitative analysis of EEG activity—to aid the diagnosis of AD—has focused on changes in activity and power across certain frequency ranges, where (Jeong, 2004) details that an increase in delta and theta alongside a decrease in alpha and beta is a standard hallmark of AD. The field is witnessing a rapid development, moving from the topological characterization of time-frequency variability to the interdependencies of such variability across densely connected networks, as in Preti et al. (2017). This development is not without its challenges, where the detection of interdependencies is susceptible to noise in EEG signals from a combination of non-cortical biologic artifacts (e.g., eye movement) and environmental noise (e.g., electrical interference), see Fitzgibbon et al. (2007). There have been significant developments in controlling and rebuilding signals in order to eliminate artifacts, but the choice of processing techniques can affect the outcome with Alam et al. (2020) noting how different algorithms produced different power spectral density for EEG frequency-bands. In this paper, we present a novel approach for reducing the impact of signal artifacts when identifying differences in the electroencephalogram (EEG) activity of those with mild cognitive impairment (MCI) and Alzheimer's disease (AD).

Functional connectivity analysis is commonly employed in neuroimaging to characterize interdependencies, often with correlation or its alternative in the frequency domain, coherence, capturing the interaction between channels. Noise and volume conduction effects can create difficulty in interpreting these measures of connectivity for EEG data, which has led to a push for improved harmonization of EEG acquisition and processing with the recommendation that connectivity measures are unreliable if considered in isolation (Prado et al., 2022). Volume conduction is particularly problematic for connectivity analysis, as many channels can become highly correlated with activity from a single source propagating through the biological tissue to be detected instantaneously by multiple channels. The imaginary part of coherency (iCOH) provides an alternative measure for connectivity that avoids the signal contamination from volume conduction, by ignoring signals with zero or π-phase lag (Nolte et al., 2004). In this way the false instantaneous activity is removed, with connectivity denoting synchronized signals with a given time lag. The removal of false connectivity by iCOH does come at a cost, as true instantaneous activity is likely erased. In this paper, we consider connectivity analysis that is robust to such removals of true activity whereby close relationships between channels can be detected without evidence of direct iCOH connectivity.

We introduce a robust methodology of EEG assessment that leverages the noise-reducing benefits of iCOH connectivity and the global analysis of eigenvector alignment (EA) to provide new perspectives on functional connectivity in working memory networks. EA was introduced by Clark et al. (2020) to study connectivity networks, from functional magnetic resonance imaging (fMRI), of patients with Alzheimer's disease. The methodology is similar to that of cosine similarity, which is applied as a data clustering metric for machine learning among other applications (Huang, 2008). For EA, the nodes—representing EEG sensors—are embedded in a Euclidean space defined by the connectivity network's dominant eigenvectors. Employing a selection of only the most dominant eigenvectors ensures that the nodes are aligned according to the network's most prominent pathways (Clark et al., 2019). Each node's placement, in this Euclidean space, depends upon every connection in the network. In this manner, eigenvector alignment is a global network assessment as a change in a single connection will affect the pattern of connectivities and so have an impact, however small, upon the alignment between all nodes.

Functional connectivity networks for memory tasks in AD have been the subject of multiple studies (Pijnenburg et al., 2004; Sperling et al., 2010). Despite this, there remains limited understanding of how AD affects the topology of working memory networks in order to impair visual short-term memory binding (VSTMB). We study VSTMB for both sporadic MCI and prodromal familial AD—caused by the single mutation E280A of PSEN1 gene (Lopera et al., 1997)—to identify the changes in network topology due to the disconnecting pathology of AD, while also considering the age-related factors that distinguish the familial and sporadic forms of AD. This work expands upon recent brain connectivity studies looking at VSTMB in AD (Parra et al., 2017; Smith et al., 2017) to build a deeper understanding of this hallmark clinical symptom of AD.

By applying EA in the study of working memory networks, we aim to improve the reliability of EEG as an assessment tool for AD with EEG, while providing a different perspective on functional connectivity networks by highlighting network structure through channel/sensor alignments. The relationship between a pair of EEG channels is usually considered by assessing the direct connectivity value, which is susceptible to noise related artifacts. In contrast, EA considers all connections and therefore—assuming the majority of connections are an accurate reflection of brain activity—a pairwise alignment should be minimally affected by a single noise artifact. There is also no need for a high direct connectivity to consider two sensors to be closely aligned according to EA. This is useful when considering connectivities derived from the imaginary part of coherency (iCOH), where instantaneous activity is erased. For example, the channels of neighboring sensors can still be closely aligned according to EA, despite a low iCOH connectivity, as the close alignment is due to similar patterns of connectivity with all other sensors.

In this paper, we shall introduce the EA methodology, including the assessment of significant alignments using random null models, before contrasting the results with established connectivity measures and providing insights on functional connectivity in working memory networks with MCI and prodromal familial AD. The main contribution of EA is that it can leverage the noise-reducing benefits of iCOH connectivity, while still identifying the network topology including relationship that are hidden by iCOH's erasure of instantaneous activity.

## 2. Materials and Methods

### 2.1. Participants and Data

In this study we investigate the “pre-dementia” phase of the disease, to consider whether the identification of brain functional abnormalities—as previously reported using connectivity analyses (Parra et al., 2017; Smith et al., 2017)—could be enhanced via the assessment of connectivity using eigenvector alignment. The mild cognitive impairment (MCI) participants are divided between familial and sporadic AD groups. The familial AD participants had developed symptoms of AD at the time of testing, but did not yet meet criteria for dementia (Acosta-Baena et al., 2011). However, dementia in these subjects will inevitably develop in due course. The sporadic AD participants hold an unknown risk for the development of dementia, but represents the most common variant. Each MCI group is compared against control participants who did not carry gene mutations and were free from psychiatric or neurological disorders.

All participants provided written informed consent in agreement with the Helsinki declaration. The Ethics Committees of the Institute of Cognitive Neurology (INECO) and University of Antioquia approved this study. Informed consent was obtained from all subjects.

#### 2.1.1. Sporadic Mild Cognitive Impairment

The subjects are 13 patients diagnosed with MCI—age 73.08 ± 9.01, education 14.08 ± 4.44, MMSE scores 26.46 ± 2.47—and 19 healthy controls—age 67.21 ± 10.14, education 16.50 ± 1.99, MMSE scores 29.50 ± 0.52—recruited from the INECO, Buenos Aires, Argentina. Criteria implemented for diagnosis derived from Petersen (2004); Winblad et al. (2004). For the sake of brevity, we only report on the MMSE. A wider clinical and neuropsychological characterization of these samples is available at Pietto et al. (2016). In summary, patients presented with a multiple-domain amnestic MCI (Acosta-Baena et al., 2011; Albert et al., 2011) as informed by Rey Auditory Verbal Learning Test (sporadic), Verbal Fluency (familial), Trail-Making Test A and B (both), and Rey Figure Copy and Recall (both). However, given the well-known limitations of MMSE for tackling subtle cognitive domains, and for generalization to cross-cultural and low-socioeconomic settings, future replication should include more extended cognitive assessments. Nine of the patients were at particularly high risk from AD conversion having been classified as single or multi-domain amnestic MCI while three classified as non-amnestic MCI multi-domain. The data consist of 128-channel EEG activity recorded with a Biosemi Active 128-channel Two system at 512 Hz and bandpass filtered from 1 to 100 Hz. This was then down-sampled to 256 Hz.

#### 2.1.2. Familial Alzheimer's Disease Dataset

The subjects are 10 patients—44.4 ± 3.2 years old (mean ± SD), years of education 7.3 ± 4.1, MMSE scores 25.20 ± 4.50—and 10 healthy controls—44.3±5.6 years old, years of education 6.8±2.9, MMSE scores 29.10 ± 1.10—from Antioquia, Colombia. Further information on these samples is available at Pietto et al. (2016), as discussed previously. Each patient carried mutation E280A of the presenilin-1 gene which guarantees early-onset familial AD. The data consist of 60-channel EEG activity recorded with a 64-channel EEG cap using SynAmps 2.5 in Neuroscan at 500 Hz and bandpass filtered from 1 to 100 Hz with impedances below 10 kΩ. Four ocular channels were discarded after being used to factor out oculomotor artifacts.

##### 2.1.2.1. Artificial dataset expansion

Establishing the similarity between the control groups from the sporadic and familial datasets is useful, given the differences in age and EEG density. The familial dataset contains a smaller sample size of 10 healthy controls, compared to the 19 recorded in the sporadic study. In the Section 3 section, these two datasets are compared directly but there is an additional comparison with an artificially expanded familial dataset. For this artificial expansion, the number of healthy controls is extended from 10 to 20 by including subject connectivity matrices twice before identifying significant differences. Such an approach is used to artificially inflate the significance of the results, and therefore is merely illustrative; used to support the direct comparison and highlight the benefit of expanding the study.

### 2.2. Visual Short-Term Memory Binding Task

EEG data was recorded while participants performed visual short-term memory binding tasks. These assess memory binding in visual short-term memory (VSTM) by contrasting performance during the recognition of colored shapes, which requires binding of shape and color in memory retention (binding), and the recognition of single shapes which only requires the retention of a constituent feature (see Parra et al., 2017 for an illustration of the task). In the shape task, where change detection assesses VSTM for shape alone, the arrays consist of three different black shapes. In the binding task, the arrays consist of three different shapes each with a different color. Each task trial consists of an encoding period (500 ms), during which a study array is displayed on screen, followed by an unfilled short delay (900 ms) and test period with a test array. During the test period, participants are prompted to respond whether or not the objects in the two arrays are identical. The positions of the objects are randomized between arrays to avoid use of location as a memory cue. Both shapes and colors are chosen randomly for each trial from sets of eight shapes and eight colors. A randomly chosen 50 percent of the trials have the same objects in both arrays. In the other 50 percent, two shapes seen during the encoding periods are replaced with two new shapes selected from the set. While in the binding task, the color of two of the shapes is changed in addition to the shape changes. All participants start with a brief practice session before undergoing one hundred trials per task. Binding and shape tasks are delivered in a counterbalanced order across participants.

### 2.3. Imaginary Part of Coherency

The functional connectivity between each pair of EEG channels is assessed from the preprocessed time series data. The coherency captures functional connectivity as it is a measure of the linear relation between two EEG channels (sensors) at a specific frequency (Khadem and Hossein-Zadeh, 2013). For channels i and j, the coherency at each frequency f is as follows:


(1) $$\begin{array}{r} {\text{COH}}_{ij}\left(f\right)=\frac{S_{ij}\left(f\right)}{\sqrt{S_{ii}\left(f\right)S_{jj}\left(f\right)}}, \end{array}$$

where *S*_*ij*_(*f*) is the cross spectrum of the signals acquired from the channel i and j, and the respective autospectra are *S*_*ii*_(*f*) and *S*_*jj*_(*f*). The imaginary part of coherency (iCOH) is the absolute value of the imaginary part, i.e., iCOH_*ij*_(*f*) = Im(COH_*ij*_(*f*)). The iCOH connectivities are calculated for four independent EEG frequency bands; delta (0.1−4 Hz), theta (4−7 Hz), alpha (7−13 Hz), and beta (13−30 Hz). The iCOH is computed using FieldTrip (Oostenveld et al., 2011)—with the multi-taper method fast Fourier transform (mtmfft) and padding equal to the trial time—to provide a 0.5 Hz resolution that is average over frequency bands. One weighted adjacency matrix was then processed for each participant undergoing each task by averaging across trials of the tasks. The use of a global network assessment for the adjacency matrix means that every connection is accounted for when determining the relationship between sensors. There is, therefore, no need to filter the adjacency matrix either through removing connections by applying a threshold or using spanning trees to construct a network topology.

### 2.4. Eigenvector Alignment

**Figure 1:**
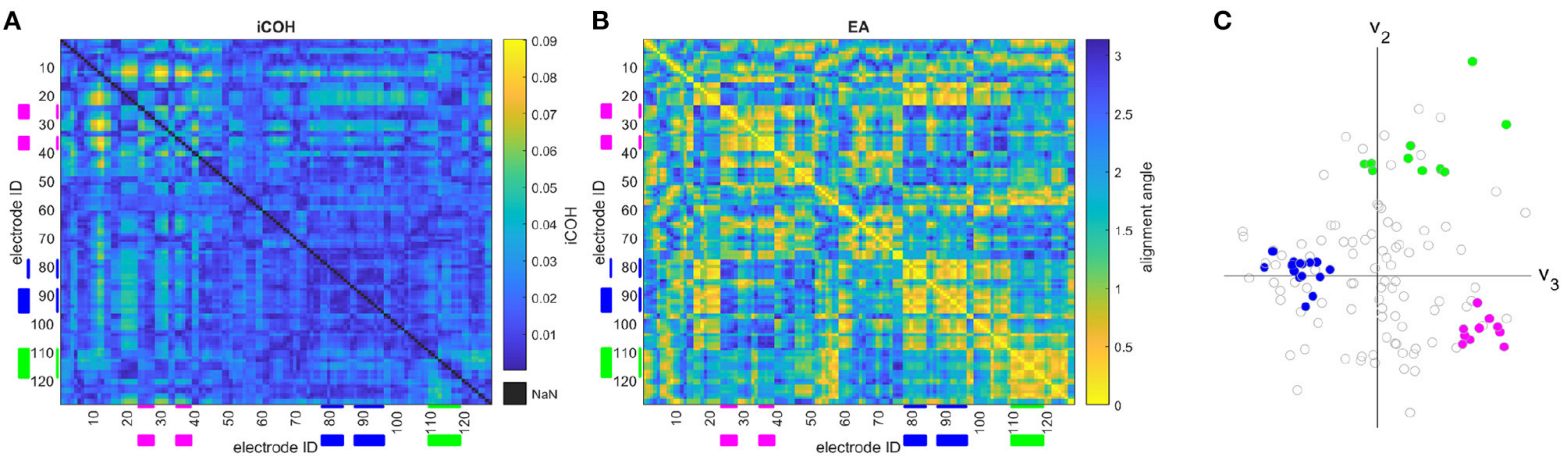
The **(A)** iCOH connectivity matrix, **(B)** eigenvector alignment matrix, and **(C)** sensor node embedding, in an eigenvector defined Euclidean space, are presented for a 128-channel EEG recording of a control subject. Color denotes node groupings in **(C)** with the related sensor IDs highlighted in **(A,B)**, where v_2_ & v_3_ are the 2nd and 3rd eigenvectors, respectively.

The alignment between sensors is evaluated, as described in Clark et al. (2020), by embedding the sensor nodes in a Euclidean space—defined by three of the system's dominant eigenvectors—and assessing the dot product of their position vectors with respect to the origin of this coordinate frame. This comparison can yield the alignment angle θ between the two vectors using the well-known relation,


(2) $$\begin{array}{c} \theta =\cos^{-1}\left(\frac{r\cdot s}{\left|r||s\right|}\right) \end{array}$$

where **r** and **s** are the position vectors for different sensor nodes in the eigenvector-defined space. Sensors are closely aligned when they have a small angle between their position vectors. In this paper, eigenvector alignment is based on the 2nd, 3rd, and 4th eigenvectors of the connectivity matrix as demonstrated in Figure 1C. The first eigenvector (Perron-Frobenius eigenvector) is not included as it is exclusively composed of non-negative entries, and therefore unable to identify network division.

Figure 1 presents an example of a subject's (Figure 1A) iCOH connectivity matrix, (Figure 1B) eigenvector alignment matrix, and (Figure 1C) sensor node embedding in an eigenvector-based Euclidean space. This figure demonstrates how the iCOH and eigenvector alignment matrices relate, which reveals that low iCOH connectivity values (e.g., between sensors 88 to 96) can still achieve small alignment angles (i.e., close alignment). Close alignment is a result of similar patterns of connectivity, as can be seen in Figure 1 for each of the highlighted sensor groupings. The three sensor groupings (marked in Figures 1A–C) demonstrate close in-group alignments, while between group alignment angles are large. This is visualized in Figure 1C, where each grouping forms a separate cluster with clear separation between groups.

#### 2.4.1. Significance Tests

Significant alignments are determined for sets of healthy control, prodromal familial AD, and sporadic MCI subjects through comparison with alignments generated by random null models. Clark et al. (2020) demonstrated that consistent alignments can be achieved when comparing against 1,000 random models, with over 90% of the same alignments recurring with separate sets of 1,000 random models. In this paper, the random models are generated by rearranging subject iCOH matrices to form randomized iCOH matrices while ensuring the same distribution of connectivity values. Each randomized iCOH matrix then being used to calculate an EA matrix.

Significance is evaluated assuming unequal variance with a t-test (Behrens-Fisher problem). Given that we are performing multiple hypothesis testing (e.g., comparison of 8128 sensor ID pairs in 128-channel EEG iCOH matrix) the p values are converted to q values by adjusting for the false discovery rate (FDR), as described by Benjamini and Hochberg (1995). A threshold of *q* < 0.05 yielding an FDR of 5% among all significant alignments.

Significant correlation and iCOH connectivities can also be evaluated in the same manner, where the randomized matrices are compared directly with the subject connectivity matrices.

#### 2.4.2. Visualizing Connections

We use a visualization of network connections throughout this paper to aid interpretation of the results. Alignments and connectivities are visualized from the top view throughout this paper with the frontal left/right and posterior left/right marked in the figures. The visualization includes a filter on the connections displayed to restrict those between both distant and neighboring sensors. This filter is particularly useful for visualizing the EA results; in general, neighboring sensors share similar connectivities to other sensors and therefore have increased alignment relative to random null models. Hence, it is more informative to look beyond these neighboring connections to reveal regions of close alignment between non-neighboring sensors. The maximum distance between sensors is also constrained as decreased alignment, with respect to random null models, is likely to occur between distant sensors. This maximum distance constraint highlights decreased alignments that occur despite the relative proximity of sensors.

It is worth noting that increased alignments are also filtered out by the maximum distance constraint. These alignments obfuscate the patterns of increased alignment, when presented in two-dimensions, so their removal is valuable when presenting results. However, these are potentially informative alignments and future work should consider what these alignments between distant sensors reveal about brain activity.

The maximum distance threshold for including connections is taken as 65% of the maximum y-axis sensor distance; 117 mm for the 128-channel coordinate positions and 104 mm for the 60-channel positions. While the minimum distance threshold is taken as 250% of the smallest distance between sensors; 50 mm for the 128-channel positions and 42 mm for the 60-channel positions. The coordinate mappings for the Biosemi Active 128-channel and 64-channel positions are given in Biosemi (2022).

#### 2.4.3. Consistent Alignments

**Figure 2:**
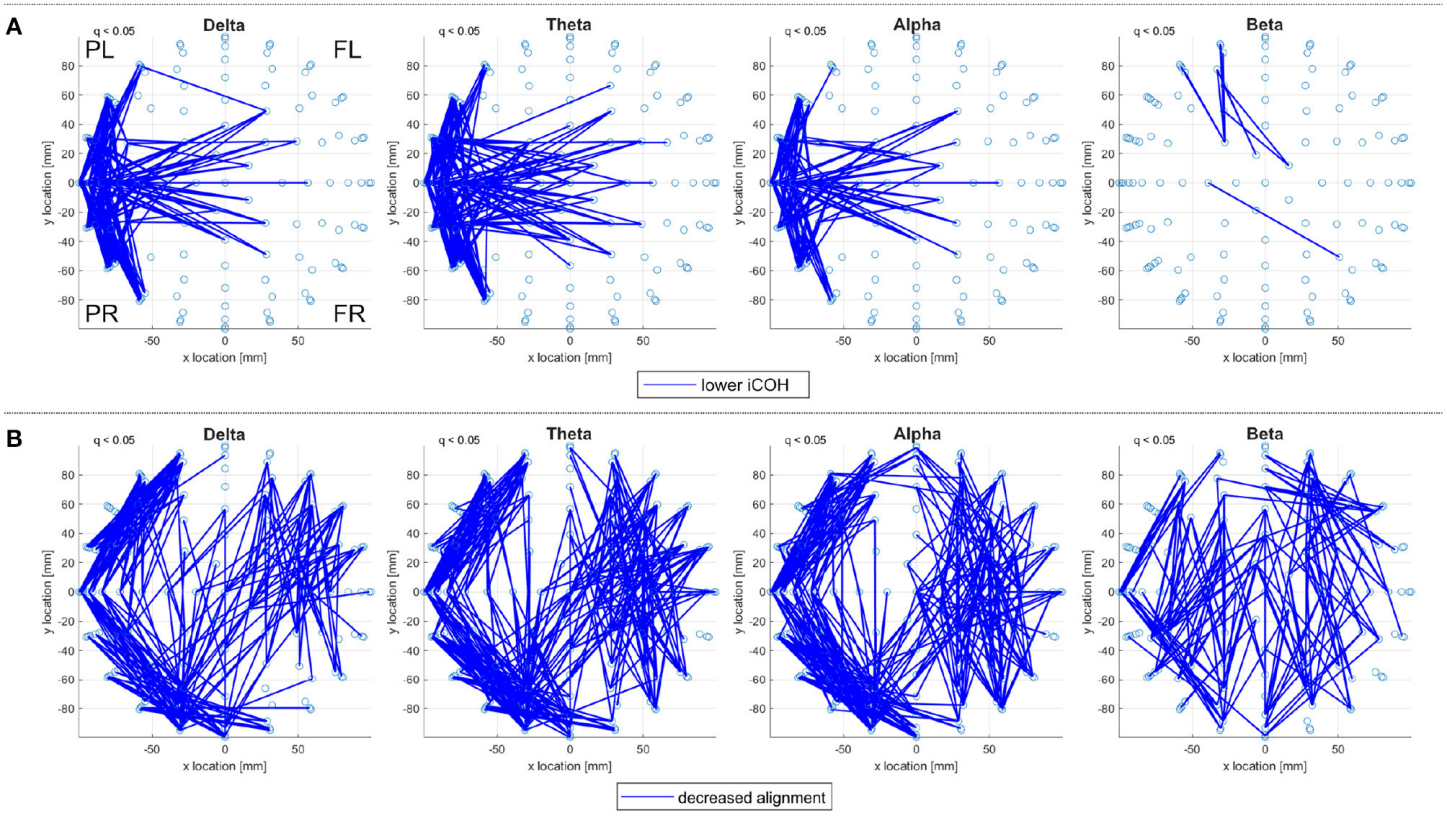
A top view of **(A)** significantly lower iCOH connectivities and **(B)** significantly decreased eigenvector alignments (EA), with respect to random null models; displayed for the delta, theta, alpha, and beta frequency ranges (left to right). Significant connections (*q* < 0.05) are identified for the set of 19 control subjects from the sporadic dataset performing the shape memory task. Only iCOH and EA connections within a defined sensor distance bound are displayed, see Section 2.4.2. The frontal left (FL), frontal right (FR), posterior left (PL), and posterior right (PR) are marked to indicate orientation.

For a given subject, there are variations in iCOH connectivities across the frequency ranges but there is also consistency especially between the delta, theta, and alpha frequency ranges. For example, Figure 2A reports consistent, significantly low, iCOH connectivities for the control subjects in the sporadic dataset. The significantly high iCOH connectivities also display similar consistency, see Supplementary Material for further detail. This consistency translates into consistent EAs when derived from iCOH, as seen in Figure 2B, where the pattern of alignments is also similar in the Beta range. Note that both correlation connectivities, generated using Pearson's correlation coefficient, and EA derived from correlation matrices differ notably between frequency ranges, for the same set of subjects performing either of the working memory tasks.

Consistency of EA between frequency ranges is not unexpected, as sensors displaying little activity at a given frequency range can still achieve close alignment if their patterns of activity are similar. Therefore EA consistency enables results to be filtered, such that only alignments appearing consistently across frequency ranges are displayed. In this paper, alignments that appear in at least three of the four frequency bands (delta, theta, alpha, and beta) are considered consistent alignments. Such an approach is useful for ensuring that reported alignments are not the product of an artifact at affecting a single frequency range.

#### 2.4.4. Glossary of EA Terminology

EA is introduced here for use in EEG functional connectivity analysis, therefore it is considered beneficial to the reader to include a glossary of the key terms.

**Table:** 

Alignment	The pairwise similarity of channel connectivities, assessed using the alignment angle.
Alignment angle	The angle between two position vectors in a Euclidean space defined by a selection of the network's dominant eigenvectors.
Increased alignment	The alignment angle between a pair of sensors is significantly smaller than alignment angles reported for the random null models.
Decreased alignment	The alignment angle between a pair of sensors is significantly larger than alignment angles reported for the random null models.
Close alignment	The alignment angle between a pair of sensors is relatively small.
Consistent alignment	A significant alignment that is reported in at least 3 of the 4 frequency bands considered (delta, theta, alpha, and beta).
Dominant eigenvector	An eigenvector associated with the largest eigenvalue of the connectivity matrix.

## 3. Results

Results shall be presented for 128-channel EEG recordings of healthy control (CON) and sporadic mild cognitive impairment (MCI) subjects, alongside 60-channel EEG recordings of healthy control (CON) and early-onset familial Alzheimer's disease (AD) subjects. Both the sporadic and familial datasets include the same working memory tasks (Shape & Bind) recorded across four frequency ranges (delta, theta, alpha, and beta).

**Figure 3:**
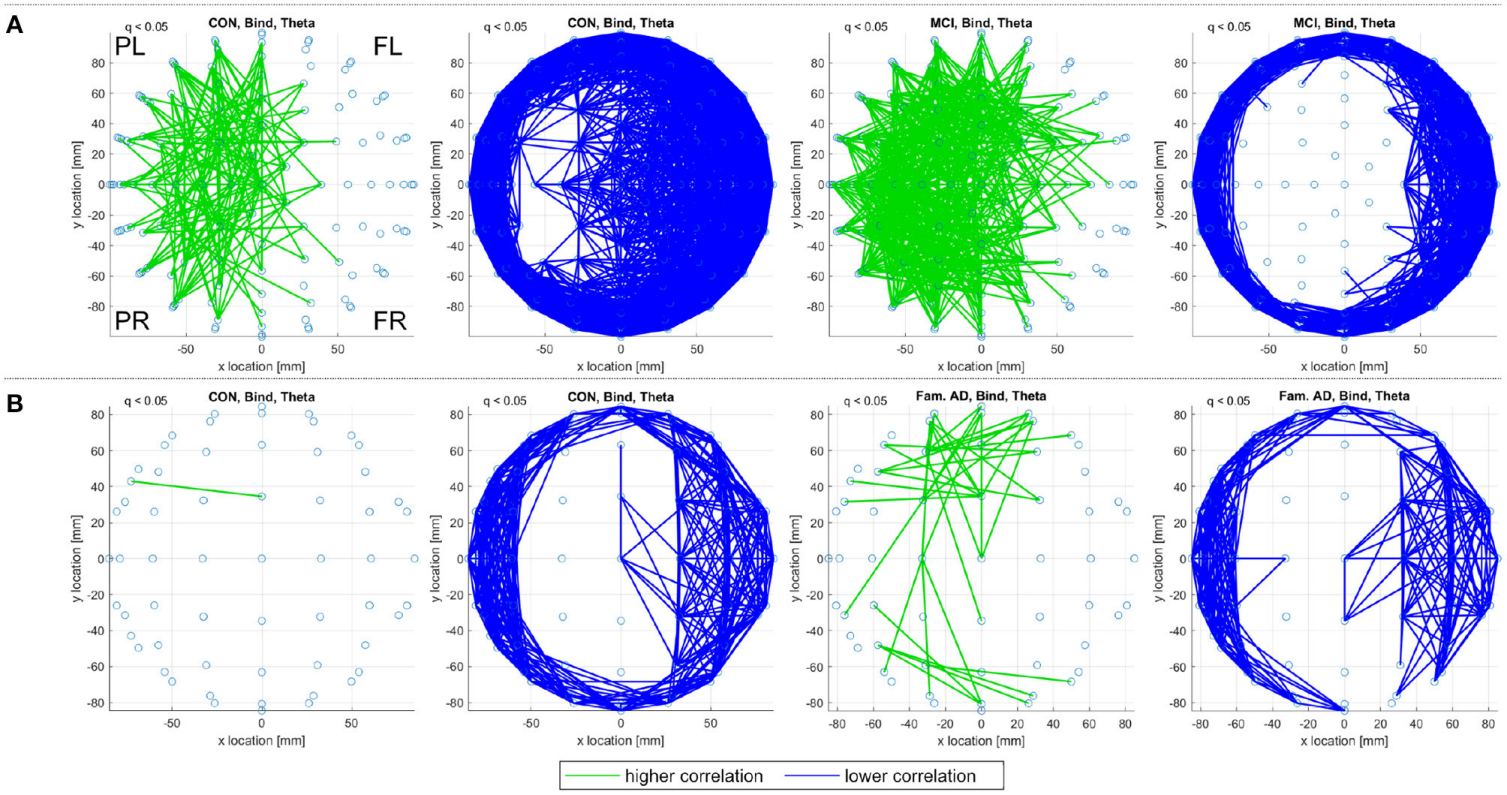
A top view of significantly higher and lower correlation (Pearson's coefficient) connectivities, with respect to random null models, for the theta frequency range. Significant correlation connections (*q* < 0.05) are identified in **(A)** for the CON and MCI subjects and in **(B)** for the CON and prodromal familial AD subjects performing the memory binding task. Only connections within a defined sensor distance bound are displayed, see Section 2.4.2. The frontal left (FL), frontal right (FR), posterior left (PL), and posterior right (PR) are marked to indicate orientation.

For *correlation* functional connectivity matrices, generated with Pearson's correlation coefficient, there are few significant connectivities detected for the delta, alpha, and beta frequency ranges in the control, sporadic MCI, or prodromal familial AD groupings. The exception is the theta frequency range where Figure 3 demonstrates clear patterns of significant connectivities that are consistent between the two datasets. In both Figures 3A,B, the control subjects display with fewer significantly higher correlation connectivities (green) and more significantly lower connectivities (blue) than the MCI and prodromal familial AD subjects for the memory binding task. A similar contrast is also observed for the shape memory task.

**Figure 4:**
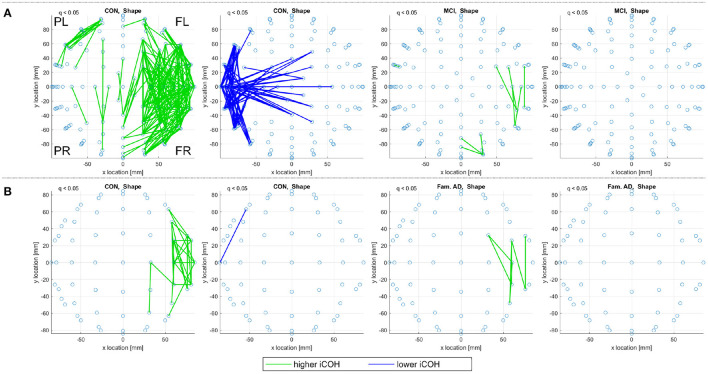
A top view of significantly higher and lower iCOH connectivities, with respect to random null models, that were consistent across at least three of the four frequency ranges investigated (delta, theta, alpha, and beta). Consistent and significant iCOH connections (*q* < 0.05) are identified in **(A)** for the CON and MCI subjects and in **(B)** for the CON and prodromal familial AD subjects performing the shape memory task. Only connections within a defined sensor distance bound are displayed, see Section 2.4.2. The frontal left (FL), frontal right (FR), posterior left (PL), and posterior right (PR) are marked to indicate orientation.

For *iCOH* functional connectivity, consistent and significant higher and lower connectivities are detected as discussed in the Section 2.4.3. These consistent connectivities are reported in Figure 4 where they indicate greater activity (higher iCOH connectivities) in the frontal regions of all subject groupings as well as lower iCOH connectivities between posterior sensors in the control groups. The control subjects present with a greater number of consistent connectivities and there are no significantly lower iCOH connectivities in either the MCI or prodromal familial AD groups.

**Figure 5:**
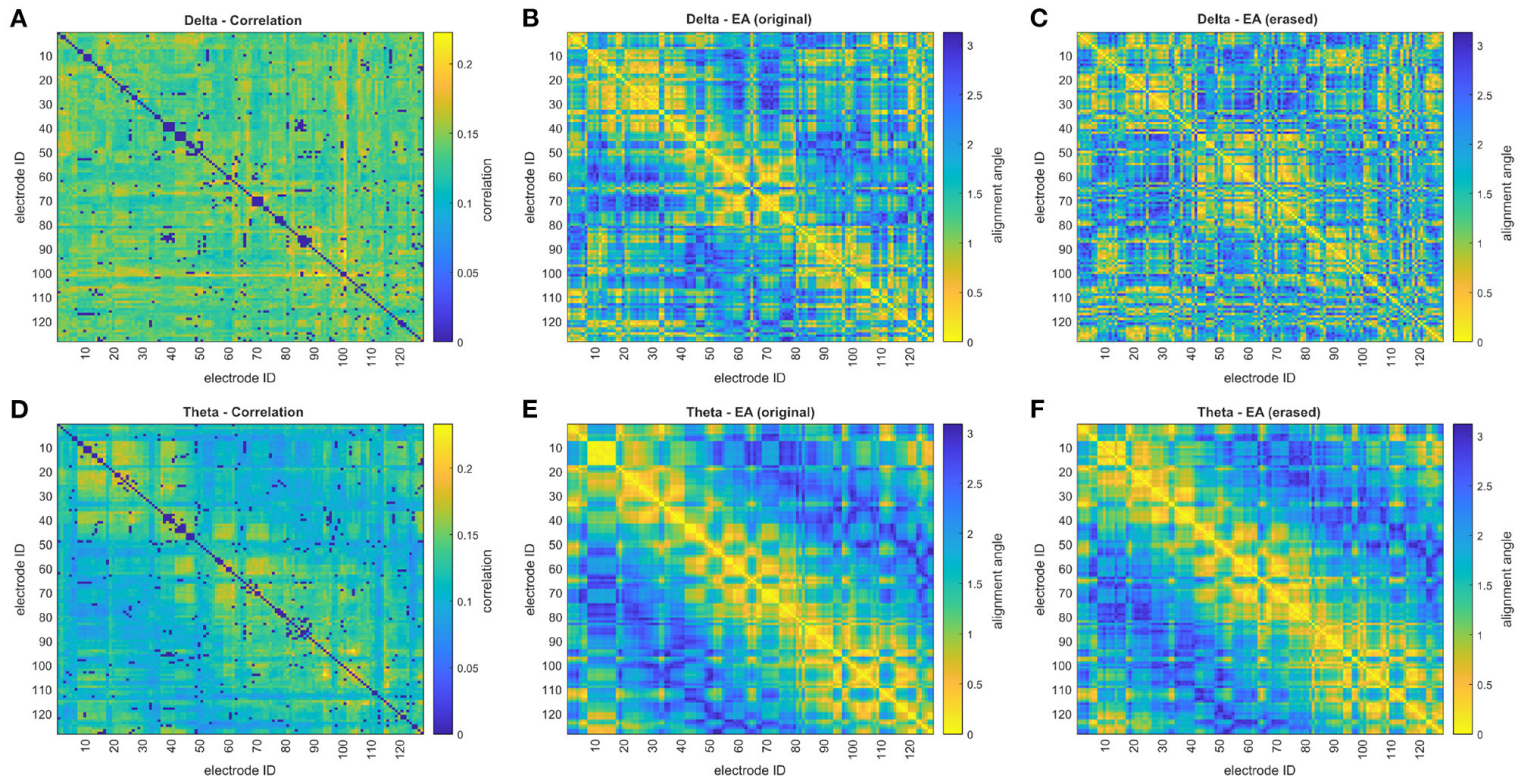
The impact of erasing correlation connections on eigenvector alignments (EA) is displayed for a control subject in the sporadic dataset. The correlation matrices, with erased connections corresponding to the 250 lowest iCOH connections (~3%) for the respective frequencies, are shown for **(A)** delta and **(D)** theta frequency ranges. The EA matrices assessed from the original (unaltered) correlation matrices are shown for **(B)** delta and **(E)** theta. The EA matrices assessed from correlation matrices, with erased connections, are shown for **(C)** delta and **(F)** theta.

For *EA* assessment of functional connectivity networks, it is envisioned that EA's reliance on the pattern of connectivities, when determining the relationship between a pair of EEG channels, will provide greater robustness to the erasure of connections than an assessment of the direct relationship. This claim is supported by Figure 5, where the effect of erasing correlation connections on EA is demonstrated for a control subject's delta and theta recordings. In both Figures 5C,F, the pattern of EA is recognizably similar to Figures 5B,E despite the removal of 250 connections (500 directional edges, just over 3% of all edges). However, it is also evident that EA is more similar for the theta recording with the mean absolute difference ΔEA = 0.16 when comparing Figure 5E and Figure 5F, while more variation is seen with the delta range, ΔEA = 0.61 when comparing Figure 5B and Figure 5C.

**Figure 6:**
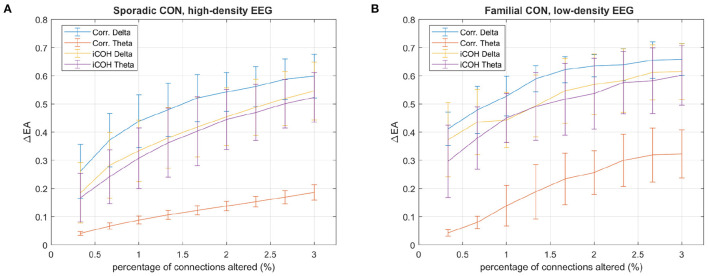
The absolute difference between EA values calculated from correlation and iCOH matrices with and without altered connections (ΔEA), against the percentage of altered connections, is presented for **(A)** high-density and **(B)** low-density EEG control recordings. Connections are altered by either erasing connections, for the correlation matrices, or creating connections, for the iCOH matrices by setting entries equal to the maximum iCOH entry. In both cases the altered connections correspond to the lowest iCOH connections for the respective frequencies. The mean ΔEA is reported for control subjects in both the familial and sporadic datasets, using the delta and theta frequency ranges recorded during the shape memory task. The standard deviation of ΔEA is denoted with error bars.

A clear distinction between delta and theta recordings in Figure 6, for the correlation matrices, confirms the observation in Figure 5 that—following connection erasure—EA is prone to greater variation with delta correlation matrices than theta. The clear pattern of correlations for theta (Figure 5D) is similar for most subjects and contributes to the presence of many significant alignments in Figure 3.

Connection erasure from correlation matrices, as in Figure 5, and connection creation in iCOH matrices are considered in Figure 6. In both cases the lowest iCOH values are altered, with correlation entries set to zero and iCOH entries replaced with the largest magnitude iCOH entry. A similar pattern is found for both the 128-channel recordings of control subjects in the sporadic dataset (Figure 6A), and the 60-channel recordings of control subjects in the familial dataset (Figure 6B). Fewer channels means fewer sensor connections, and Figure 6 shows that this results in less robustness of EA with greater variation from the 60-channel data for the same percentage of erased connections. In general, the theta iCOH matrices produce less EA variation after alteration but the difference is far less notable than it is for the correlation matrices. Note that the robustness of EA to alterations in connectivity, for the alpha and beta frequency ranges, are similar to those reported for delta.

**Figure 7:**
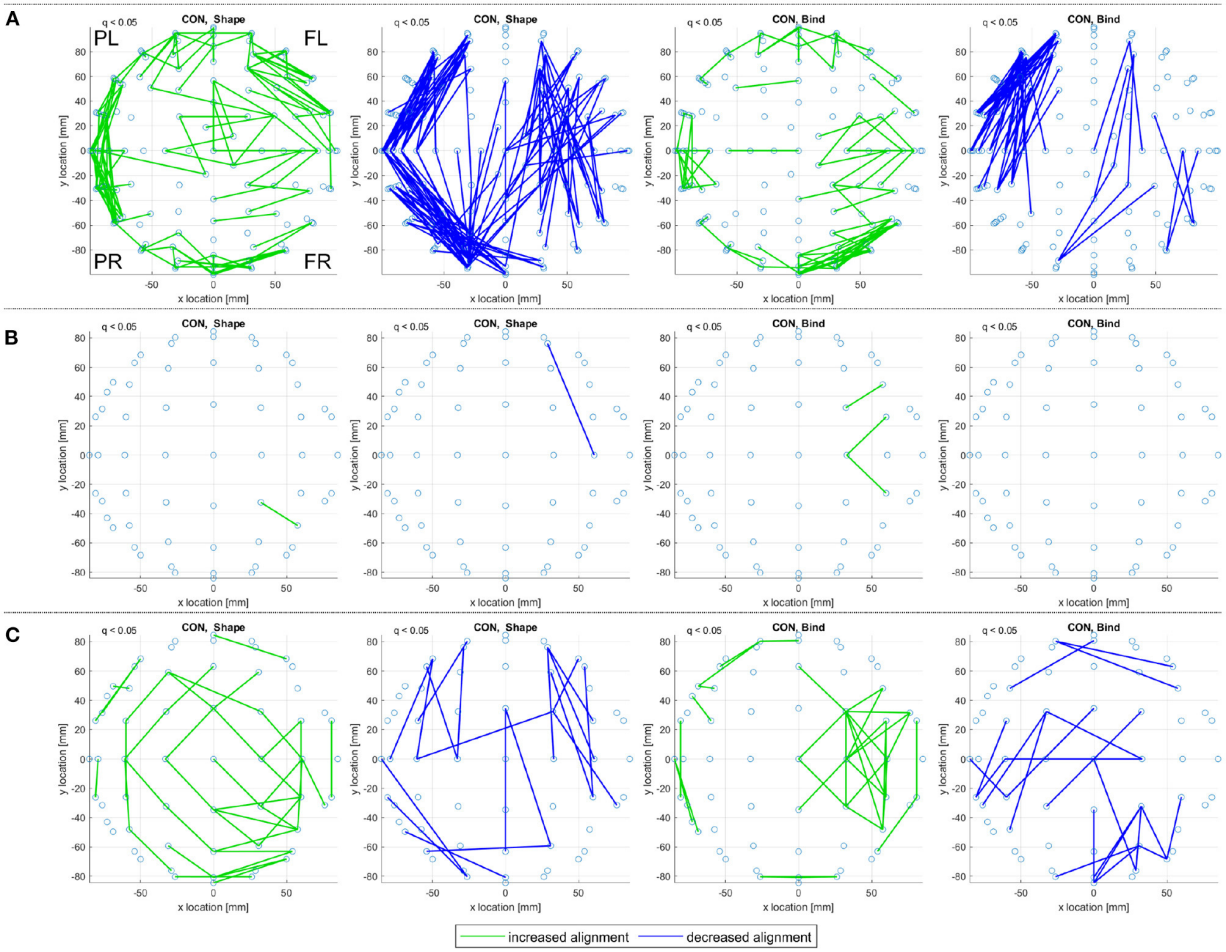
A top view of significantly increased and decreased eigenvector alignments, with respect to random null models, are displayed if an alignment is consistent across at least three of the four frequency ranges investigated (delta, theta, alpha, and beta). These consistent and significant (*q* < 0.05) EAs are shown for the set of control subjects in **(A)** the sporadic (128-channel) and **(B)**, **(C)** the familial (60-channel) datasets for both the shape memory (shape) and memory binding (bind) tasks. In **(C)** the set of control subjects is artificially expanded by duplicating each subjects scan results. Only EA connections within a defined distance bound are displayed, see Section 2.4.2. The frontal left (FL), frontal right (FR), posterior left (PL), and posterior right (PR) are marked to indicate orientation.

The consistent alignments detected across frequency ranges (see Consistent Alignments) are displayed in Figure 7 for the control subjects in both the sporadic and familial datasets.

In Figures 7A, 19 control subjects are recorded with 128-channel EEG while in Figure 7B only 10 control subjects are recorded with 60-channel EEG. Figure 7B displays far fewer consistent alignments than Figure 7A, which highlights the impact of using both a smaller dataset and a lower density recording. It is possible to gain an insight on the influence of fewer channels on the alignments detect by analyzing only a subset of the available channels. Reducing the sporadic dataset (high-density, 128 channel, recordings) down to a 32 channel setup (as defined in Biosemi, 2022) produces more alignments than the 60 channel results in Figure 7B but far fewer than in the original analysis. Therefore, it is not density alone that is at fault for the loss of significant alignments in the familial AD dataset. However, taking a smaller subset of 16 sensors for the sporadic dataset does highlight that EA is sensitive to density as only a few alignments are detected for the control subjects and none recorded for the MCI subjects. For more details see the Supplementary Material.

The comparison between the alignments detected for both control groups in Figure 7 can be aided by artificially extending the dataset through subject duplication, see Section 2.1.2.1. Figure 7C, therefore, considers an artificially expanded dataset of 20 low-density recordings with the artificial inflation of significance resulting in more evident patterns of alignment. The artificial extension of the familial dataset reduces the reliability of the results in Figure 7C, but it is useful in highlighting commonality between the sporadic and familial control groups. The control groups are separated by both EEG density and age, where the sporadic are 67.21 ± 10.14 years old and the familial are 44.3 ± 5.6 years old. Despite these differences, the pattern of decreased alignments for the shape memory task (CON, Shape) in Figures 7A,C are very similar. There are also notable similarities in the increased alignments observed in the memory binding task (CON, Bind) where the increased alignments are more concentrated between the frontal sensors. The similarities are not as obvious with either the increased alignments in the shape memory task or the decreased alignments in the memory binding task. Given the polarity of the relationship between increased and decreased alignments, similarity in one between control groups suggests that the other is unlikely to be contradictory.

**Figure 8:**
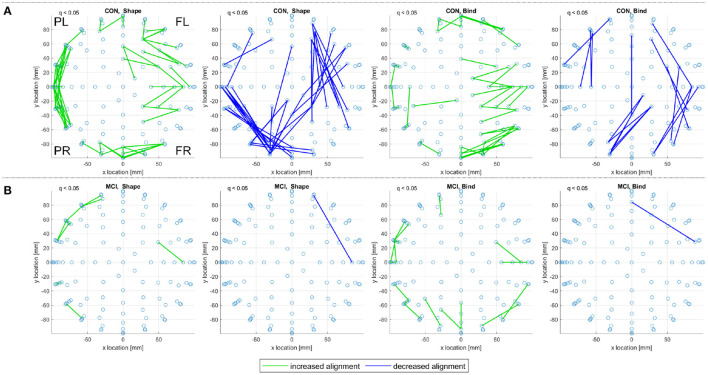
For **(A)** the CON and **(B)** the MCI groups in the sporadic dataset, a top view of consistent alignments, both increased and decreased alignments with respect to random null models, are displayed. These consistent alignments (*q* < 0.05) are shown for both the shape memory (shape) and memory binding (bind) tasks. Only EA connections within a defined sensor distance bound are displayed, see Section 2.4.2. The frontal left (FL), frontal right (FR), posterior left (PL), and posterior right (PR) are marked to indicate orientation.

In Figure 8, consistent alignments are compared between control and sporadic MCI subjects. Unlike Figure 7 only a subset of 13 control subjects—to match the number of MCI subjects—are used to identify consistent alignments. Figure 8 still presents a stark contrast between the alignments detected for control and MCI subjects, where MCI subjects display fewer consistent alignments but similar patterns of alignment with the main distinction being the location of posterior left and right *increased alignments* in the shape test (MCI, Shape).

**Figure 9:**
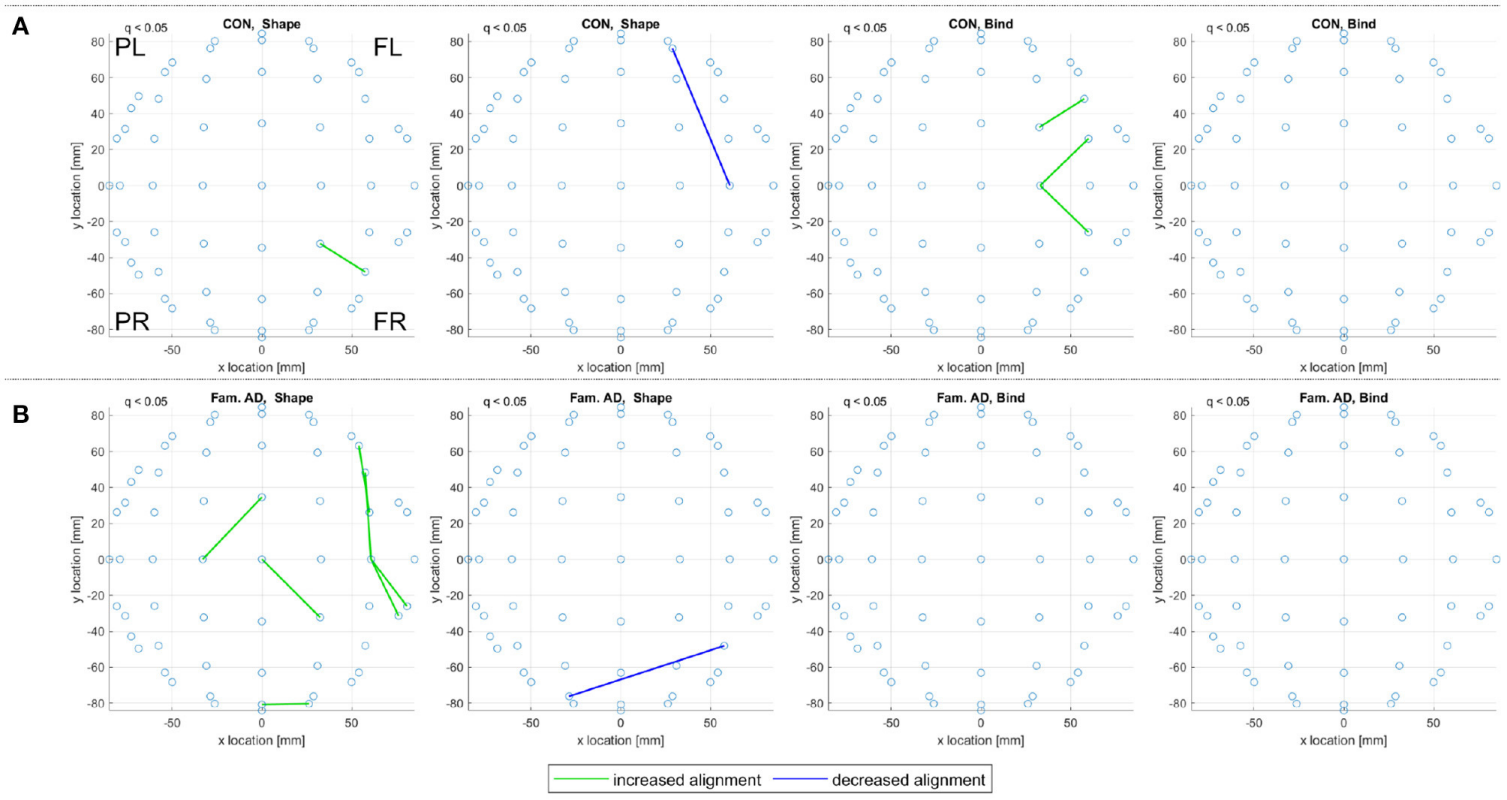
For **(A)** the CON and **(B)** the prodromal familial AD (Fam. AD) groups in the familial dataset, a top view of consistent alignments, both increased and decreased alignments with respect to random null models, are displayed. These consistent alignments (*q* < 0.05) EAs are shown for both the shape memory (shape) and memory binding (bind) tasks. Only EA connections within a defined sensor distance bound are displayed, see Section 2.4.2. The frontal left (FL), frontal right (FR), posterior left (PL), and posterior right (PR) are marked to indicate orientation.

Figure 9 reports on control and prodromal familial AD subjects, where only a few consistent alignments are detected. The location of consistent alignments, reported in Figure 9B for the familial AD subjects, differ from both sets of control subjects in the sporadic and familial datasets. In particular, for the familial AD, shape memory task (Fam. AD, Shape) there is a greater concentration of increased alignments in the frontal region (+x location) and a decreased alignment along the right side (-y location) of the brain (from channels CP6 to F4, i.e., centroparietal to frontal). These differences in alignment locations are highlighted clearly when reviewing the significant alignments for each frequency range, included in Supplementary Material. There are no consistent alignments for the familial AD, memory binding task (Fam. AD, Bind), but increased alignments in Figure 9A for control subjects (CON, Bind).

## 4. Discussion

By introducing a methodology, combining eigenvector alignment and iCOH connectivity, for the study of EEG functional connectivity networks, we have sought to provide a different perspective on sensor connectivity networks with a robust analysis that can lead to improved detection and intervention for AD.

Both correlation and iCOH connectivity results report differences between control and MCI/prodromal familial AD subjects. In both Figure 3 (theta frequency range correlations) and Figure 4 (consistent iCOH connectivities across frequency ranges) the location of connectivities remains similar for each subject group with the main difference being the density of significant connectivities. A similar result is observed for the consistent alignments of sporadic MCI subjects, where fewer alignments are found but they present in similar locations to their healthy control counterparts. In contrast, eigenvector alignment (EA) derived from iCOH matrices indicates a different network topology for prodromal familial AD subjects performing the shape memory task (Figure 9) than is seen with either control group or the sporadic MCI subjects.

A strength of our sample design is the availability of prodromal familial AD participants who had not yet developed symptoms of AD at the time of testing, but will inevitably do so in due course. The opportunity to contrast such data with that obtained from equivalent sporadic AD participants is unique as the latter group holds unknown risk for the development of dementia and yet represents the most common variant. The difference in alignments for prodromal familial AD subjects is seen primarily for the shape memory task, as there are no consistent alignments reported for the memory binding task. The sparse presentation of alignments, highlights that confidence in the results would be improved by expanding the sample size, to include more subjects, or by increasing recording density. However, the breakdown of significant alignments across the separate frequency ranges, included in Supplementary Material, reveals that the decreased alignment reported in Figure 9B is not a lone artifact but rather part of a pattern of decreased alignments on the right side (-y location) for the Familial AD, shape test, results. These decreased alignments report disconnection between the right centroparietal (CP)/temporal (T) channel locations with the right anterior frontal (AF)/frontal (F) locations (channels CP6 to F4 in Figure 9B). This is in contrast to the patterns reported in Figures 7–9A where decreased alignments in the shape test are seen between the occipital (O) locations and bilateral centroparietal electrodes, as well as between the left frontocentral (FC) and middle (*y* = 0) frontal/frontocentral electrodes (see Biosemi, 2022 for EEG channel layouts).

Some demographic and clinical features of our samples are worth considering as they can aid the interpretation of our results. The age discrepancies should be noted. Groups entering analysis of sporadic MCI were over 65 years of age while those entering analysis of familial MCI were in their 40's. Age has proved a confound when it comes to early detection of sporadic later-onset AD (Parra, 2022). Most neurocognitive changes associated to MCI linked to Alzheimer's disease are also present in normal aging though to a lesser extent. Accrued wisdom suggests that these early changes are linked to increased activity (i.e., functional reorganization) aimed at compensating for the impact of the underlying neuropathology. Parra et al. (2011) compared samples of sporadic and familiar AD (the latter from the same cohort as considered herein) and have shown that behaviorally these are indistinguishable. Pietto et al. (2016) later explored similar hypothesis using EEG and behavioral measures in MCI subjects reporting very similar findings (i.e., shared neurocognitive phenotypes across disease variants). Such shared features across clinical samples of different ages can reflect age-related compensatory changes which have been well-documented by other studies (Parra et al., 2013; Reuter-Lorenz and Park, 2014; Cabeza et al., 2018). We therefore feel compelled to suggests that our findings from familial AD cohorts can inform on neurocognitive changes linked to the disease mechanisms which are unaccounted for by aging (see also Pietto et al., 2016). Future studies will be needed to replicate these across larger samples of cases from populations affected by different diseases variants and mutations.

By assessing pairwise relationships with EA, it is envisioned that the impact of noise in the recording and the erasure of true instantaneous activity (zero or π-phase) by iCOH processing will be reduced. Figure 1 demonstrated how close alignments (i.e., small alignment angles) can be associated with the lowest iCOH connectivities. This confirms that the erasure of connectivity between a pair of channels does not prevent the detection of close alignments, as long as the pattern of connectivity elsewhere is similar for both channels. The extent to which EA is robust to connectivity removal is explored in Figures 5, 6 by monitoring the change in the EA values after removal or creation of connectivity connections. The altered connectivities were those with the lowest iCOH values, for a given frequency range, and are therefore likely to include erased true connectivities. EA is found to be most robust when considering theta range correlation matrices, especially for alterations to correlation matrices. In comparison to other frequency ranges, theta correlations display clearer delineation between high and low connectivities that creates distinctive patterns of connectivity for each channel. This clear structure enables the derived EAs to remain similar even after the removal of hundreds of connectivities, as evidenced by low variation in EA values in Figure 5. EA is effective when combined with iCOH, which is known to mitigate against volume conduction, as it can still identify the topological structure of EEG functional connectivity networks despite connectivity erasure.

The study of EA robustness to connectivity removal also highlights that high-density, 128-channel, recordings are more robust than low-density, 60-channel, recordings for the same percentage of erased connections. This contributes to the lack of detected alignments (consistent and significant) from low-density recordings, where using a subset of 32 and 16 channels from the 128-channel recording dataset was found to produce notably fewer consistent alignments. Results with few consistent alignments indicating that either no common connectivity patterns were present or too few subjects were considered to produce significant findings. The low-density dataset is artificially extended by duplicating subject data, in order to form a viable comparison with the high-density recordings. This limits the reliability of detected alignments, but more similarities can now be seen in the consistent and significant alignments detected for low- and high-density control groups. This supports the reliability of EA analysis across differing EEG setups, but an expanded dataset is required to validate this finding.

The results from the 128-channel EEG recordings in Figure 8 present a case for the use of EA with iCOH to identify clear differences in brain activity between healthy controls and subjects with MCI, at high-risk of developing sporadic AD. The differing numbers of CON and MCI subjects were confirmed in Figure 8 to have only a marginal effect on the difference in significant alignments. Therefore, it can be concluded that the patterns of EEG activity are more consistent for CON subjects than MCI. A similar finding has been reported in Clark et al. (2020) where notably fewer alignments were found for AD subjects than health controls from functional magnetic resonance imaging scans.

## Data Availability Statement

The raw data supporting the conclusions of this article will be made available by the authors, without undue reservation.

## Ethics Statement

The studies involving human participants were reviewed and approved by the Institute of Cognitive Neurology (INECO) and University of Antioquia. The patients/participants provided their written informed consent to participate in this study.

## Author Contributions

RC, KS, JE, and MP contributed to the conception and design of the study. RC wrote the first draft of the manuscript and developed the analysis methodology. AI and MP led the data collection. All authors contributed to manuscript revision, read, and approved the submitted version.

## Funding

The work was supported by Alzheimer's Society grants ASR42303 and AS-SF-14-008 awarded to MAP in collaboration with AI and JE. MP's and AI's EEG work was currently supported by BrainLat Seed Research Grant Program (BL-SRGP2020-02). AI was supported by grant from Takeda CW2680521; CONICET; FONCYT-PICT (2017-1818, 2017-1820); ANID/FONDECYT Regular (1210195, 1210176, 1220995); ANID/FONDAP (15150012); ANID/PIA/ANILLOS ACT210096; and the Multi-Partner Consortium to Expand Dementia Research in Latin America (ReDLat), funded by the National Institutes of Aging of the National Institutes of Health under award number R01AG057234, an Alzheimer's Association grant (SG-20-725707-ReDLat), the Rainwater Foundation, and the Global Brain Health Institute.

## Conflict of Interest

The authors declare that the research was conducted in the absence of any commercial or financial relationships that could be construed as a potential conflict of interest.

## Publisher's Note

All claims expressed in this article are solely those of the authors and do not necessarily represent those of their affiliated organizations, or those of the publisher, the editors and the reviewers. Any product that may be evaluated in this article, or claim that may be made by its manufacturer, is not guaranteed or endorsed by the publisher.
