# Supplementary material for: Robust Assessment of EEG Connectivity Patterns in Mild Cognitive Impairment and Alzheimer's Disease
Source: Front Neuroimaging. 2022 Jul 11;1:924811. doi: 10.3389/fnimg.2022.924811 (PMC10406240; doi:10.3389/fnimg.2022.924811)
Supplement: Supplementary file 1 [file Data_Sheet_1.PDF]

Supplementary Material: Robust assessment of EEG connectivity patterns in Mild Cognitive Impairment and Alzheimer's disease

Fig. S1 presents a visualisation of the iCOH matrices for a single control subject in the familial (high-density EEG) dataset. Commonality in the iCOH connectivity patterns is apparent across most of the frequency ranges for the separate working memory tasks, but the intensity of values can be seen to differ. This commonality translates into significantly lower and higher iCOH connectivities than found in random null models, as reported in Fig. S2. By displaying the separate frequency ranges, for the healthy control (Fig. S2 (**A**)) and sporadic MCI subjects (Fig. S2 (**B**)) it is possible to see that although the number of significant connectivities differ, there is no obvious difference in the location of significant connectivities.

In Fig. S3,S4,S5, & S6 significant eigenvector alignments are reported for the delta, theta, alpha, & beta frequency ranges. In the paper, these results are primarily displayed by visualising only the consistent alignments that appear in at least 3 of the 4 ranges. By displaying the results for each frequency range, the patterns of alignment are clearer in cases where few consistent alignments are detected such as Fig. S5 & S6. The similarity between decreased alignment patterns of the sporadic and familial healthy control subjects can be more clearly seen by contrasting the significant alignments across frequency ranges. In particular, the decreased alignment locations in Fig. S5 (**A**) reflect the three regions of decreased alignment seen repeatedly in Fig. S3 (**A**). In the main paper the prodromal familial AD subjects presented with a different pattern of decreased alignment to any of the other subject groupings with decreased alignment along the right side (-y locations). This pattern is even more evident when considering the separate frequency ranges where the majority of decreased alignments are located on the right side of the brain in Fig. S6 (**B**).

One of the differences between the sporadic and familial dataset was the use of high- and low-density EEG, respectively. There was discussion about the impact of EEG density on the alignments results. Therefore, alignments are presented in Fig. S7 & S8 by taking the sporadic dataset (originally 128 channel) and reducing it to use only a subset of 32 and 16 channels, respectively. These results reveal a reduction in alignments from 128 channels down to 32 in Fig. S7 and a further reduction in alignments for 16 channels in Fig. S8. When using 32 channels the alignments are still recognisably similar to those reported for the control subjects from the 128 channel recordings, but EA appears sensitive to the recording density according to these results. Future studies can consider what is the optimal EEG setup, in terms of density, and whether EA can detect clear alignment trends in low-density recordings by increasing the normal and clinical samples.

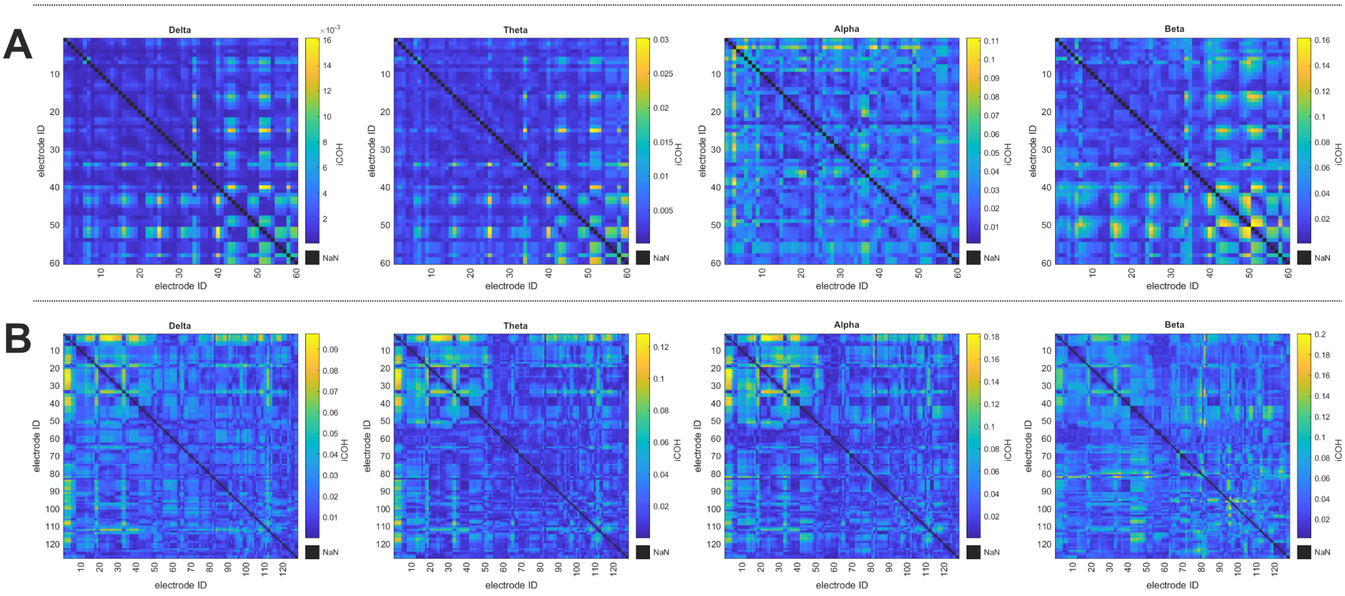

Figure S1. iCOH connectivity matrices for a control subjects in familial dataset (lew-density EEG); delta, theta, alpha, and beta frequency ranges are displayed (left to right) for **(A)** the shape memory task and **(B)** the memory binding task recordings.

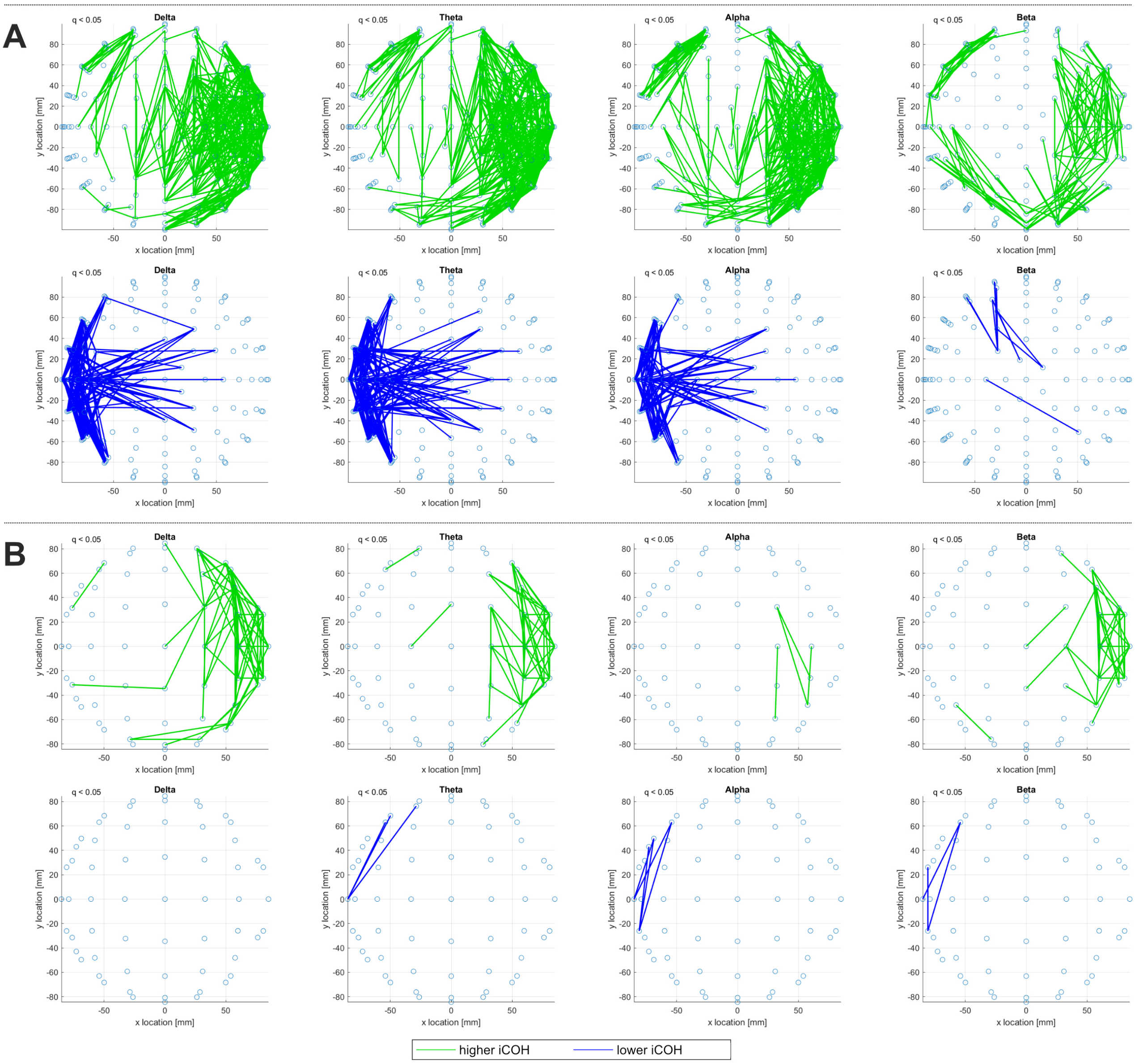

Figure S2. Shape memory task, sporadic dataset, iCOH; top view of significantly higher and lower iCOH connectivities ($q < 0.05$), with respect to random null models, displayed for frequency ranges delta, theta, alpha, and beta. Connectivities are detailed for **(A)** 19 healthy control subjects and **(B)** 13 sporadic MCI subjects, where only iCOH connections within a defined electrode distance bound are displayed.

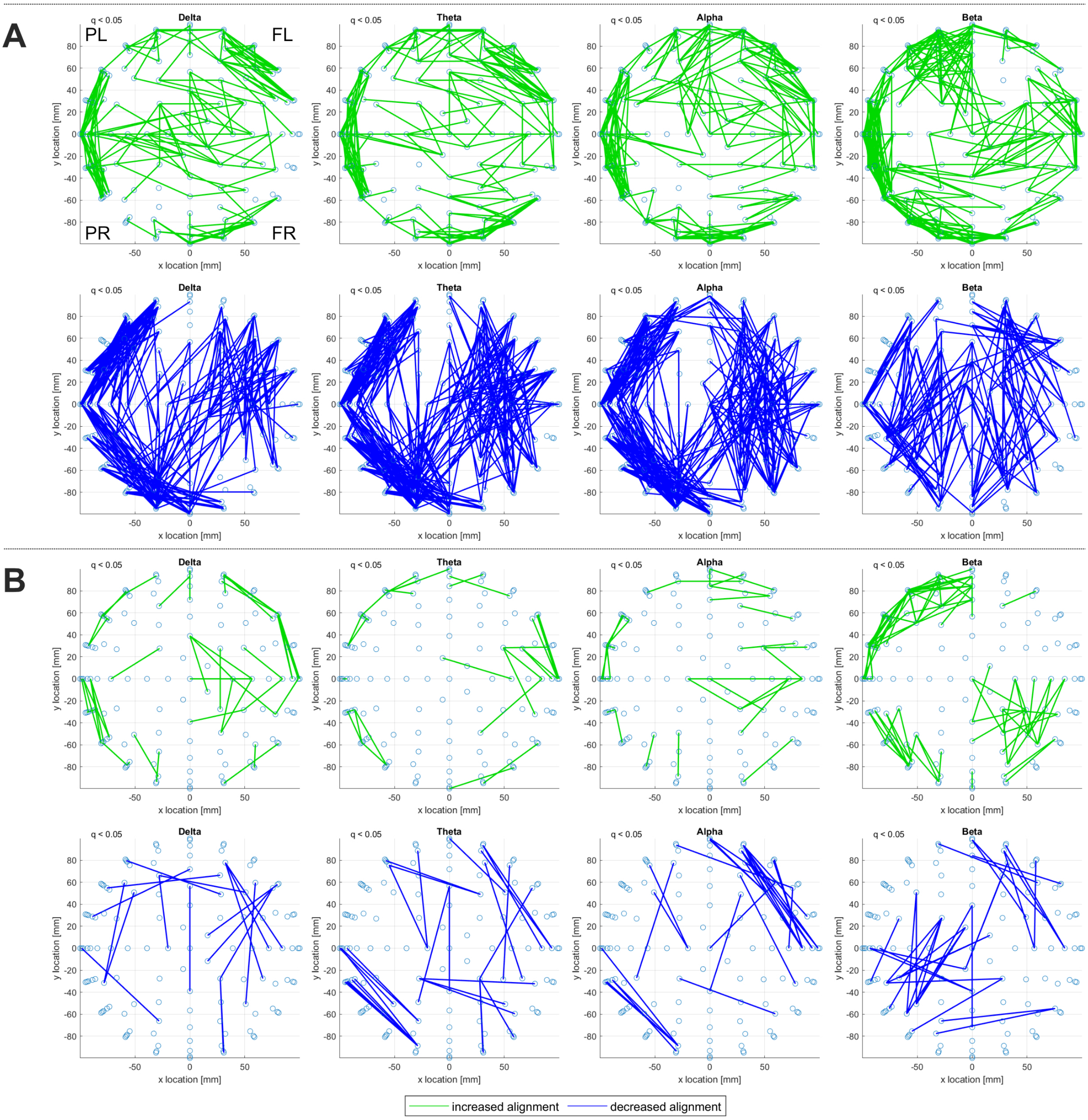

Figure S3. Shape memory task, sporadic dataset, EA; top view of significantly increased and decreased alignments ($q < 0.05$), with respect to random null models, displayed for frequency ranges delta, theta, alpha, and beta. Alignments are detailed for **(A)** 19 healthy control subjects and **(B)** 13 sporadic MCI subjects, where only EA connections within a defined electrode distance bound are displayed.

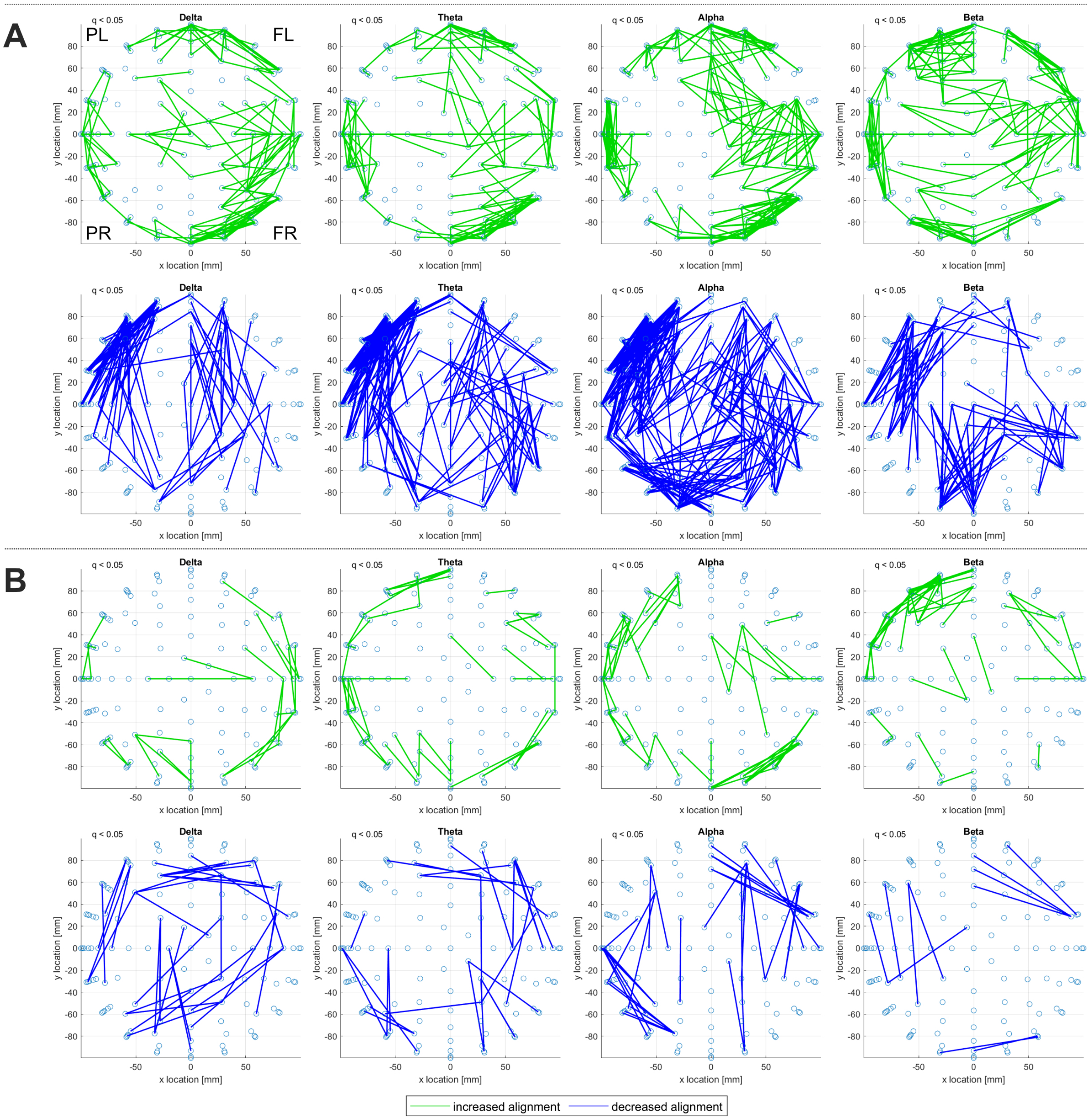

Figure S4. Memory binding task, sporadic dataset, EA; top view of significantly increased and decreased alignments ($q < 0.05$), with respect to random null models, displayed for frequency ranges delta, theta, alpha, and beta. Alignments are detailed for **(A)** 19 healthy control subjects and **(B)** 13 sporadic MCI subjects, where only EA connections within a defined electrode distance bound are displayed.

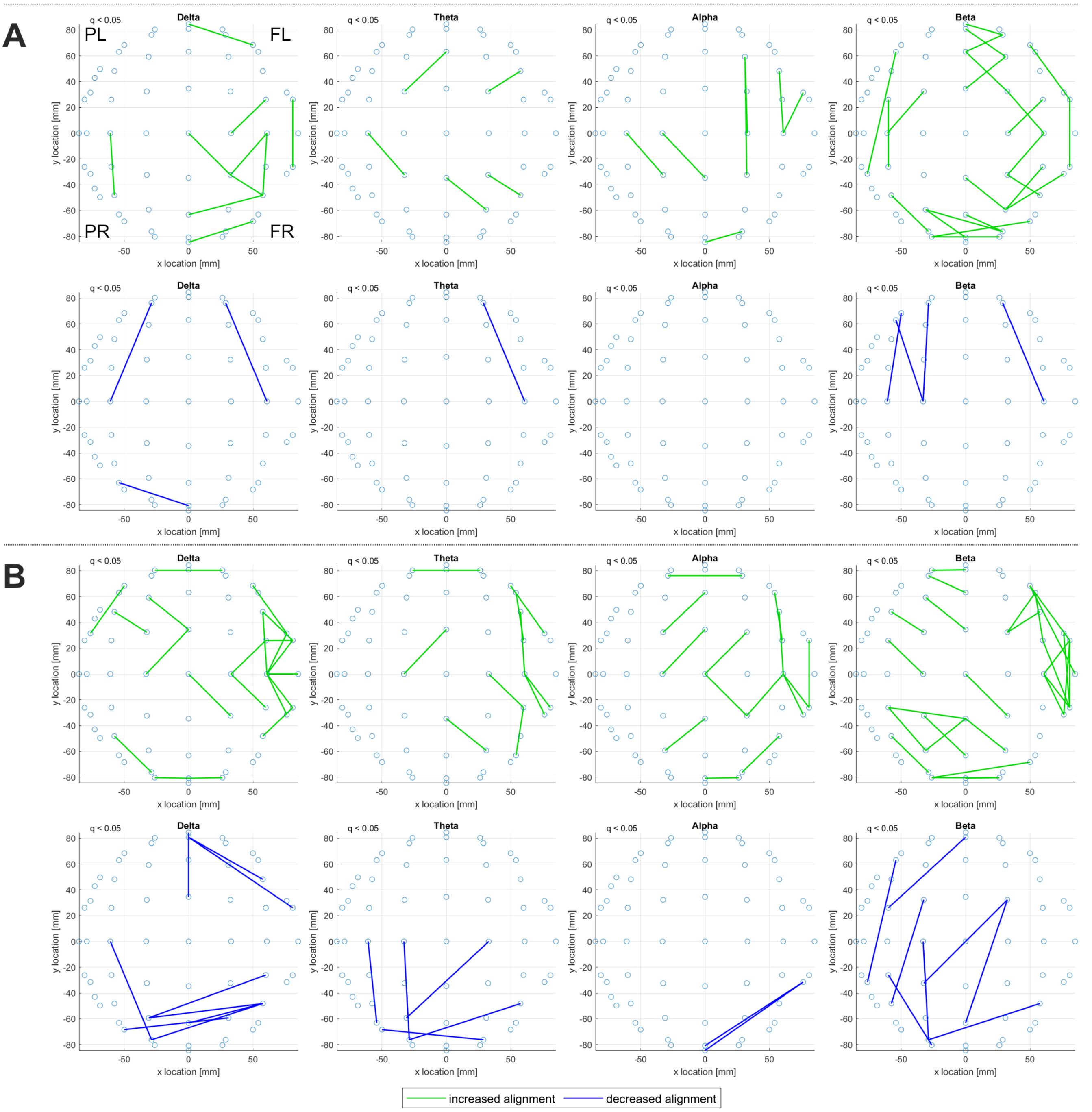

Figure S5. Shape memory task, familial dataset, EA; top view of significantly increased and decreased alignments ($q < 0.05$), with respect to random null models, displayed for frequency ranges delta, theta, alpha, and beta. Alignments are detailed for **(A)** 10 healthy control subjects and **(B)** 10 prodromal familial AD subjects, where only EA connections within a defined electrode distance bound are displayed.

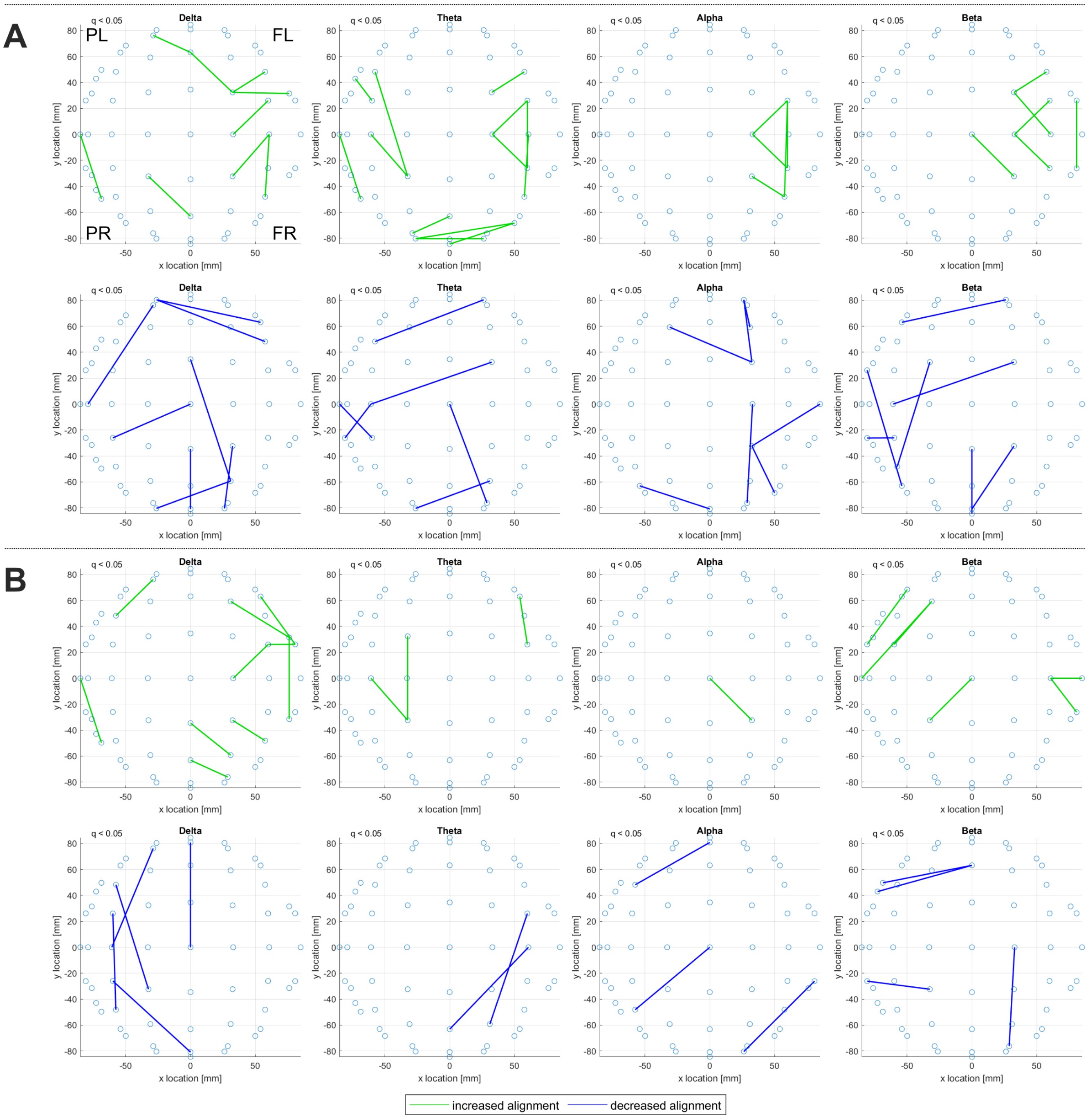

Figure S6. Memory binding task, familial dataset, EA; top view of Significantly increased and decreased alignments ($q < 0.05$), with respect to random null models, displayed for frequency ranges delta, theta, alpha, and beta. Alignments are detailed for **(A)** 10 healthy control subjects and **(B)** 10 prodromal familial AD subjects, where only EA connections within a defined electrode distance bound are displayed.

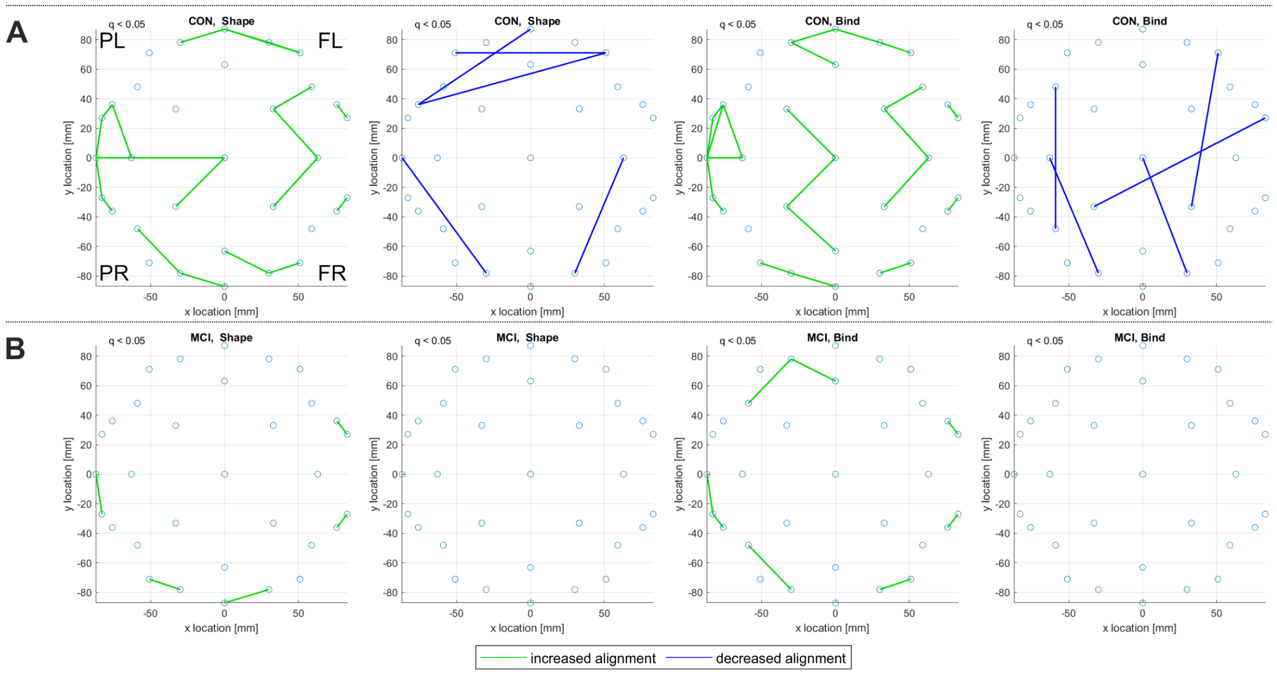

Figure S7. For a subset of 32 channels (from 128) the consistent alignments from the (**A**) CON and (**B**) MCI groups in the sporadic dataset are displayed. A top view presents both increased and decreased alignments with respect to random null models. These consistent alignments ($q < 0.05$) are shown for both the shape memory (shape) and memory binding (bind) tasks. The frontal left (FL), frontal right (FR), posterior left (PL), and posterior right (PR) are marked to indicate orientation.

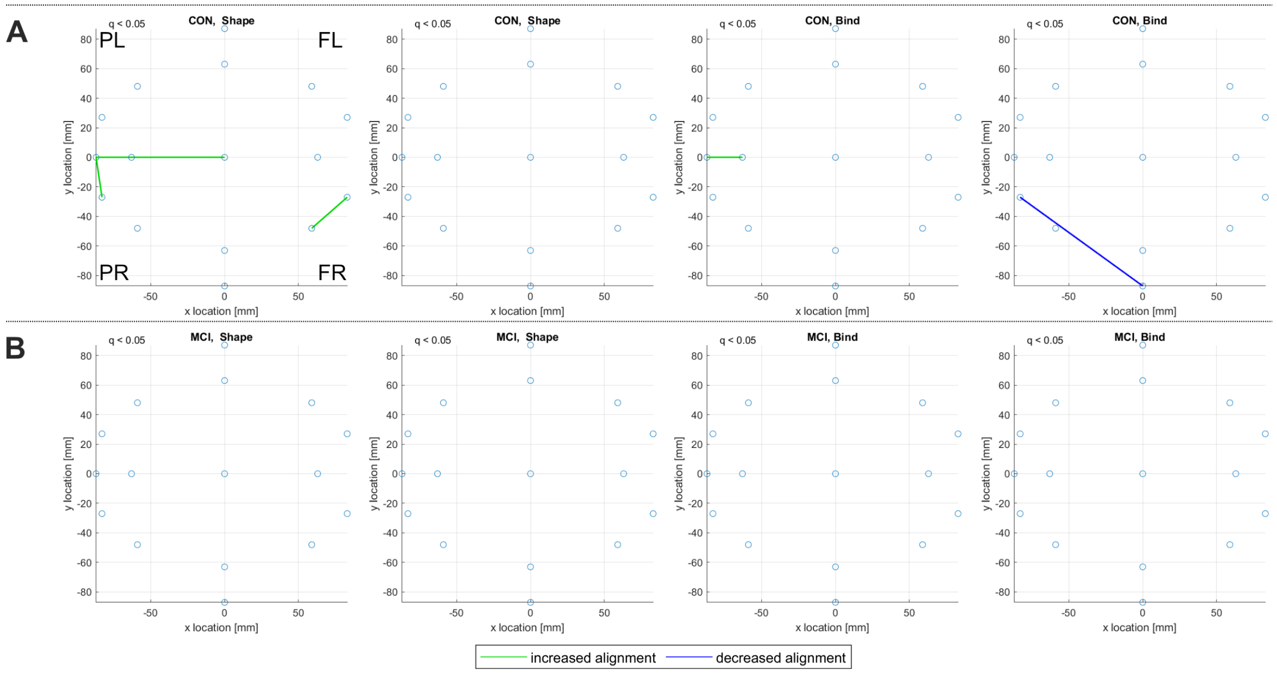

Figure S8. For a subset of 16 channels (from 128) the consistent alignments from the (**A**) CON and (**B**) MCI groups in the sporadic dataset are displayed. A top view presents both increased and decreased alignments with respect to random null models. These consistent alignments ($q < 0.05$) are shown for both the shape memory (shape) and memory binding (bind) tasks. The frontal left (FL), frontal right (FR), posterior left (PL), and posterior right (PR) are marked to indicate orientation.
